# Size of living space as a moderator for central and peripheral refractions in children

**DOI:** 10.1038/s41598-023-37454-w

**Published:** 2023-07-04

**Authors:** Kai Yip Choi, Tsz Wing Leung, Henry Ho-Lung Chan

**Affiliations:** 1 Centre for Myopia Research, School of Optometry, The Hong Kong Polytechnic University, 11 Yuk Choi Road, Hung Hom, Kowloon, Hong Kong; 2 Centre for Eye and Vision Research (CEVR), 17W Hong Kong Science Park, Pak Shek Kok, Shatin, Hong Kong; 3 Research Centre for SHARP Vision (RCSV), The Hong Kong Polytechnic University, 11 Yuk Choi Road, Hung Hom, Kowloon, Hong Kong

**Keywords:** Environmental social sciences, Risk factors, Public health, Epidemiology

## Abstract

Undesirable living environment may impose risk on myopia development. Furthermore, peripheral refractive error was suggested to contribute to juvenile eye growth modulation. This study aimed to investigate the interaction between peripheral refractive error and living environment in relation to central refractive status in Hong Kong schoolchildren. Central and peripheral refractive errors, axial length (AL), and corneal radius of curvature (CR) were measured in 573 schoolchildren (age 9.5 ± 0.9 years). The AL/CR ratio was used to represent the central refractive status, accounting for non-cycloplegic refraction. The relative peripheral refractive errors (RPRE) up to ± 20° eccentricities were converted into power vectors: spherical-equivalent error (SER) and J_0_ astigmatic components and fitted with quadratic equations. The second-order coefficients of SER (a_SER_) and J_0_ astigmatism (a_J0_) and home size reported by parental questionnaires were analyzed to indicate their relationships with AL/CR. Our results showed that children with higher AL/CR lived in smaller homes (*p* = 0.01) and had a more hyperopic (*p* < 0.001) but less astigmatic RPRE (*p* = 0.01). We further analyzed the relationship between AL/CR with RPRE for children living in small (< 300 ft^2^), moderate (300–600 ft^2^), and large home sizes (> 600 ft^2^). Regardless of the home size, a higher AL/CR remained moderately correlated with a more hyperopic a_SER_ (all *p* < 0.001). However, a higher AL/CR was associated with a more positive a_J0_ only in children living in large homes, and the relationships were not significant for small and moderate home sizes. Linear regression models further indicated that home size was a significant moderator contributing to the relationship between AL/CR and a_J0_. In conclusion, our results were consistent with previous studies, showing that children with axial myopia usually lived in smaller homes and had more hyperopic defocus and more positive J_0_ astigmatism. However, the relationship between peripheral astigmatism and axial refraction was modulated by the home size of Hong Kong schoolchildren. While peripheral astigmatism is hypothesized as a visual cue for axial refractive development in children, extrinsic environmental factors, such as home size, might interfere with the relationship and dominate refractive development.

## Introduction

Short-sightedness, or myopia, has been a global epidemic affecting billions^1^, and the prevalence is still rising^2^. Myopes, especially those with high myopia, are subject to irreversible vision loss^3^, leading to reduced productivity and quality of life^4^.

Environmental factors have consistently been addressed in the myopia literature, for instance the characteristics of the indoor and outdoor scenes^5^. In particular, the crowdedness and urbanicity of the living environment were associated with juvenile refractive error. The Sydney Myopia study reported that children living close to the city center, where the population density is higher, had a higher prevalence and degree of myopia^6^. Also, a flat-style type of housing in Sydney, as well as a taller residential building in China^7^, were associated with more myopia. A higher population density and smaller home size were also associated with a more myopic refractive error in Hong Kong^8^. Urbanization and limited living space are risk factors for myopia in the modern world.

The contribution of peripheral refractive errors to myopia development has attracted considerable attention over the past decades. Over the decades, studies reported an association between axial myopia and relative peripheral hyperopia^9–11^. These studies raised the question of whether visual inputs from the central, peripheral, or entire retina would be utilized and contribute to modulation of juvenile eye growth. Emerging evidence from animal studies indicates that the peripheral retina, rather than only the fovea centralis, plays a critical role. For examples, the peripheral retina was able to compensate for localized blurred signals by modulating regional eye growth^12,13^. In addition, even after eliminating the foveal input by laser-ablation, the eye was still able to detect the imposed optical defocus and modulate the growth towards the focal plane^14^. In clinical trials, several optical interventions showed promising myopia control effects by inducing peripheral myopic defocus to bring the focal plane in front of the retina^15–18^. Despite the convincing evidence from animal studies and clinical trials, the results from longitudinal observational studies failed to establish a solid causal relationship between the baseline peripheral refractive error and the subsequent myopia progression in children^11,19,20^. The current study addressed the gap in our understanding of the relationship between peripheral refractive error and myopia development. Specifically, we hypothesized that additional factors should be considered to elucidate the role of peripheral refractive error on myopia development.

Myopia is a multifactorial condition influenced by intrinsic and extrinsic factors, including peripheral refractive error and living environment. However, the interplay between these factors remains unclear. Our previous study has shown that children living in small homes are at a higher risk of developing myopia^8,21^, possibly because the living environment can introduce a different dioptric profile to the eye that might interact with the intrinsic peripheral refractive errors^22^. Our previous longitudinal study demonstrated that peripheral refractive error alone was not sufficient to predict myopia progression. However, after considering the environmental dioptric profile, peripheral refractive error became a significant contributor to myopia development. Building on these findings, we hypothesized that the impact of intrinsic peripheral refractive error on myopia progression would be inhibited by extrinsic environmental factors. Specifically, weak extrinsic environmental stimulation would increase the importance of intrinsic peripheral refractive error, while dominant environmental influences would diminish its role. However, our previous study was limited by a small sample (n = 50). To test this hypothesis, the current study examined Chinese schoolchildren’s peripheral refractive error stratified by the size of living space. Our aim was to elucidate the interaction between these intrinsic and extrinsic factors in modulating myopia, with a particular focus on the differential effects of peripheral refractive error and home sizes. Our results would provide valuable insights into the mechanisms underlying myopia and contribute to the development of future interventions.

## Materials and methods

### Study design and population

This study was conducted in five Hong Kong local primary schools by random-cluster sampling stratified according to the population density due to its potential association with the myopia^6,8^: two schools from low (< 10 k persons/km^2^) and mid (10–30 k persons/km^2^), respectively, and one school from high (> 30 k persons/km^2^) population density regions. A total of 635 children participated, of whom 43 were excluded because of abnormal visual acuity (pinhole visual acuity worse than LogMAR 0.0 equivalent, n = 3), corneal opacity (n = 1), strabismus (n = 6), or receiving myopia control intervention, such as orthokeratology, progressive lenses, or dual-focus contact lenses (n = 33). An additional 19 children were excluded because their pupils were too small for peripheral refraction. Therefore, 573 of them had completed peripheral refraction up to 20° eccentricity and were included for analysis. Their age and refractive status were comparable with the excluded data (independent t-test, all *p* > 0.35). The experimental procedures followed the tenets of the Declaration of Helsinki and were approved by the Human Subjects Ethics Sub-committee of The Hong Kong Polytechnic University. Informed consent and written assent were obtained from the parents/legal guardians and subjects, respectively.

### Data collection procedures

Central and peripheral refractive errors were measured using an open-field autorefractor (Shin-Nippon, NVision K5001, Japan) operated by a registered optometrist at the school campus during school teaching hours. No cycloplegic agent was instilled to avoid disturbing children’s classroom learning. Subjects were instructed to binocularly fixate at Maltese crosses (angular size: 2.4°) located at the central, ± 10°, and ± 20° eccentricities along the horizontal visual field at a viewing distance of 6 m. The selected central field size was based on the findings of previous studies indicating these retinal regions were the most responsive to optical defocus^23–25^, as well as avoiding the location of the optic nerve head (i.e., the blind spot) at approximately 15° eccentricity of the temporal visual field^26^. Subjects were asked to keep their head stationary on the headrest and turn their eyes to the distant fixation targets during central and peripheral objective refractions. Refraction was carried out only after the subject achieved steady fixation as monitored through the display of the autorefractor. For each subject, the whole measurement at all five eccentricities (i.e., nasal 20°, nasal 10°, central, temporal 10°, and temporal 20° fields) was completed within 2 min. Measurements were repeated until five repeatable readings (± 0.50 DS and ± 0.50 DC) for each field location were obtained. The on-axis axial length (AL) and corneal radius of curvature (CR) were measured using an optical biometer (IOLMaster, Carl Zeiss Meditec, Germany) operated by a trained ophthalmic assistant. Five measurements with a signal-to-noise ratio > 2 were taken. The averaged refraction and ocular biometry data were used for analyses.

### Data processing and statistical analysis

Refractive errors obtained from autorefraction were decomposed into vector components, i.e., the spherical-equivalent refraction (SER), J_0_, and J_45_ astigmatic components^27^:

$$\mathrm{SER}=\mathrm{S}+\frac{\mathrm{C}}{2}$$

$${\mathrm{J}}_{0}=-\left(\frac{\mathrm{C}}{2}\right)\mathrm{cos}2\alpha$$

$${\mathrm{J}}_{45}= -\left(\frac{C}{2}\right)\mathrm{sin}2\alpha$$

where S is the spherical power, C is the cylindrical power, and α is the axis of the negative sphero-cylindrical form ($S+C\times \alpha$). SER indicates the dioptric position of the circle of least confusion. Positive J_0_ indicates the tendency of with-the-rule astigmatism while negative J_0_ indicates the tendency of against-the-rule astigmatism. J_45_ indicates the tendency of oblique astigmatism, by which a positive value indicates tendency towards 45° meridian while negative value indicates tendency towards 135° meridian. Relative peripheral refractive error (RPRE) was calculated by subtracting the vector components of the central field from the peripheral fields. Although the accommodation was not pharmacologically controlled, it was reported to have minimal effect on the RPRE profile.

The refractive profiles of SER and J_0_ along the horizontal visual field were modeled using a quadratic equation, ${a(Eccentricity-b)}^{2}+c$, with the built-in “LINEST” function in Microsoft Excel (Microsoft 365, Redmond, WA, USA). The resulting second-order coefficients were obtained (i.e., a_SER_ and a_J0_) and were used to quantify the change of refractive errors across the visual field^10,28^. A negative second-order coefficient indicates a more myopic blur for a_SER_ and more against-the-rule astigmatism for a_J0_, while positive second-order coefficients reveal more hyperopic defocus and with-the-rule astigmatism. Due to the low magnitude, J_45_ was omitted in the analysis. For the present analysis, the first- (b) and zero-order (c) coefficients, which indicated the symmetry and y-intercept of the refractive profile, were excluded, as our focus was to characterize the variation of refractive profile across eccentricities.

In this study, non-cycloplegic refraction was used to assess children’s central spherical refractive status, but uncontrolled accommodation might over-estimate the magnitude of myopia. Therefore, axial length to corneal radius of curvature (AL/CR) ratio was chosen as the primary outcome instead of non-cycloplegic SER. This decision was made because AL/CR ratio was strongly associated with cycloplegic SER^29^ and independent to ocular accommodation^30^. In addition to AL, which is correlated with myopia resulting from excessive elongation of the eyeball, AL/CR ratio also accounts for the variations in corneal power among emmetropic and ametropic eyes^29,30^. Consequently, AL/CR ratio provides a more comprehensive measure of central spherical refractive status. Non-cycloplegic SER and AL were used as secondary outcomes to supplement the refractive and ocular biometrical findings.

All statistical procedures were performed using SPSS (IBM, ver. 22, United States). As data from right and left eyes were strongly correlated, only the data from the right eye were presented in this study. The AL/CR, AL, and SER were compared across three home size groups: < 300 ft^2^, 300–600 ft^2^, and > 600 ft^2^, obtained from a parental questionnaire, using one-way analysis of co-variance (ANCOVA) controlled for age. The age-controlled relationship between central refractive and biometric status (i.e., AL/CR, AL, and SER) and the second-order coefficients of peripheral refractive errors (i.e., a_SER_ and a_J0_) was analyzed in regression analyses (Variance inflation factors < 2.0) stratified by home size. A moderator term was created to investigate the interaction effect between home size and the second-order coefficients as the primary analysis. In addition, a Fisher’s R-to-Z transformation, followed by a χ^2^-test for heterogeneity, was applied to compare the age-controlled partial correlation coefficients for central and peripheral refractive errors in each home size group as a secondary analysis.

## Results

### Demographics, refraction, and relationship with home size

The demographic information, refractive status, and ocular biometry of the subjects are shown in Table 1. Subjects were stratified into three home size groups. While no significant differences in age (F_2,570_ = 0.80, *p* = 0.45) and the proportion of gender (χ^2^_2,570_ = 1.48, *p* = 0.48) were found among the three groups, home size had a significant aged-adjusted effect on AL/CR (F_2,570_ = 4.46, *p* = 0.01) and SER (F_2,570_ = 7.77, *p* < 0.001) as previously reported, except AL (F_2,570_ = 2.60, *p* = 0.08) which could be due to a reduced statistical power owing to a smaller sample size than the previous study. For astigmatism at the central field, home size was neither associated with J_0_ (One-way ANCOVA, *p* = 0.06) or J_45_ (One-way ANCOVA, *p* = 0.39) after controlled for age.

**Table 1:** Demographic information and ocular parameters for the home size groups.

Gender (N)	All	Home size (ft^2^)			
		< 300	300–600	> 600	χ^2^ (*p*)
*Male*	297	83	157	57	1.48 (0.48)
*Female*	276	89	141	46	

Age (year)	All	Home size (ft^2^)			
		< 300	300–600	> 600	ANOVA F (*p*)
	9.5 ± 0.9	9.6 ± 0.9	9.5 ± 0.9	9.6 ± 0.8	0.80 (0.45)

Ocular biometry	All	Home size (ft^2^)			
		< 300	300–600	> 600	Age-controlled ANCOVA F (*p*)
*Axial length (AL, mm)*	23.66 ± 1.04	23.70 ± 1.13	23.70 ± 1.05	23.46 ± 0.79	2.60 (0.08)
*Mean corneal curvature (CR, mm)*	7.78 ± 0.25	7.76 ± 0.28	7.78 ± 0.24	7.79 ± 0.26	0.36 (0.70)
*AL/CR ratio*	3.05 ± 0.12	3.05 ± 0.13	3.05 ± 0.13	3.01 ± 0.10	4.46 (0.01)

Axial refractive error	All	Home size (ft^2^)			
		< 300	300–600	> 600	Age-controlled ANCOVA F (*p*)
*SER (D)*	− 1.13 ± 1.74	− 1.34 ± 1.91	− 1.21 ± 1.78	− 0.55 ± 1.12	7.77 (< 0.001)
*J*_*0*_* astigmatic component (D)*	0.23 ± 0.37	0.22 ± 0.35	0.26 ± 0.40	0.17 ± 0.31	2.81 (0.06)
*J*_*45*_* astigmatic component (D)*	0.01 ± 0.21	0.00 ± 0.20	0.02 ± 0.20	0.02 ± 0.24	0.95 (0.39)

Data are presented as mean ± standard deviation.

*SER* spherical equivalent refraction.

### Peripheral refractive profiles in myopes and non-myopes

**Figure 1:**
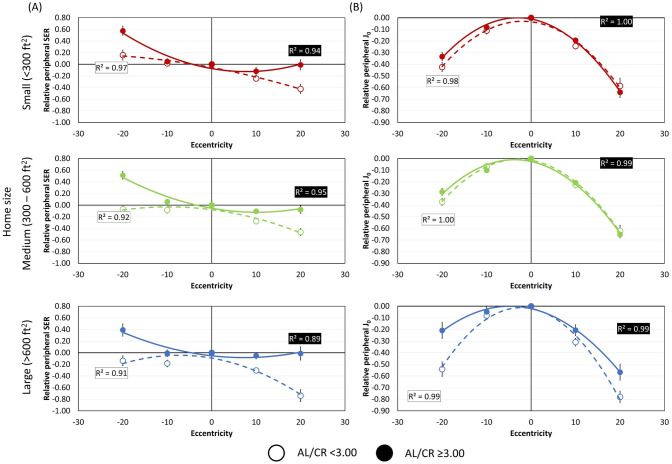
Peripheral refractive profiles stratified by home size. (**A**) SER. (**B**) J0. Open symbols represent AL/CR < 3.00 while filled symbols represent AL/CR ≥ 3.00.

Regardless of the home size, peripheral refractive profile alone was different in children with and without myopia. Figure 1 shows the respective peripheral refractive profile for children with AL/CR < 3.00 versus those with AL/CR ≥ 3.00, corresponding to approximately − 0.38 D of SER (Supplementary Fig. S3) stratified by home sizes. Consistent with previous reports, a more hyperopic peripheral refractive error was observed in children with a more myopic axial refractive error in all home sizes. Particularly, peripheral SER was significantly more myopic in children with AL/CR < 3.00 (a_SER_ − 0.51 ± 1.29) than those with AL/CR ≥ 3.00 (a_SER_ 0.71 ± 1.57, t = − 10.12, *p* < 0.001). On the other hand, differences for peripheral J_0_ was less prominent in children with AL/CR < 3.00 (a_J0_ − 1.29 ± 0.89) compared with those with AL/CR ≥ 3.00 (a_J0_ − 1.11 ± 0.80, t = − 2.52, *p* = 0.01).

### Peripheral refractive profile and home size

In multiple regression analyses controlled with age, all the second-order coefficients were significantly correlated with AL/CR (Table 2), in which a higher AL/CR was moderately associated with a more hyperopic and weakly associated with a less astigmatic blur. A similar trend was also observed in AL and SER (Supplementary Tables S1 and S2).

**Table 2:** Regression and correlation statistics stratified by home size groups for AL/CR.

Regression coefficients − unstandardized B (95% confidence interval)	Home size (ft^2^)				Moderator t (*p*)
	All	< 300	300–600	> 600	
a_SER_	0.04 (0.03, 0.04)	0.04 (0.03, 0.05)	0.04 (0.03, 0.05)	0.03 (0.02, 0.04)	− 0.39 (0.69)
a_J0_	0.02 (0.01, 0.04)	0.01 (− 0.01, 0.03)	0.02 (0.00, 0.04)	0.05 (0.03, 0.07)	**2.42 (0.02)**

Partial correlation coefficients − r (*p*)	Home size (ft^2^)				χ^2^ (*p*)
	All	< 300	300–600	> 600	
a_SER_	0.50 (< 0.001)	0.43 (< 0.001)	0.51 (< 0.001)	0.55 (< 0.001)	1.85 (0.40)
a_J0_	0.16 (< 0.001)	0.07 (0.40)	0.12 (0.03)	0.47 (< 0.001)	**13.96 (0.001)**

Bolded text indicates significant interaction effect.

**Figure 2:**
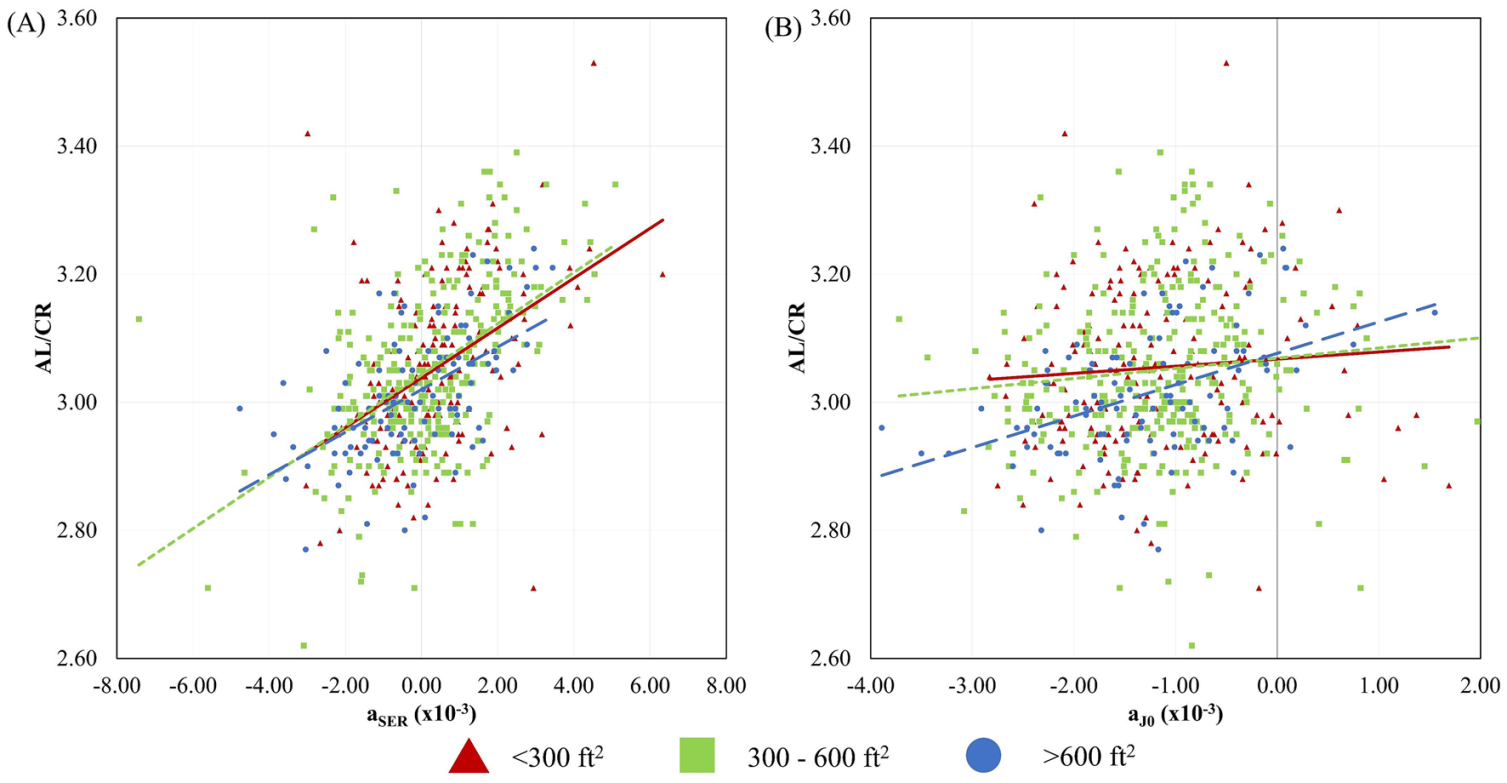
Relationships between AL/CR ratio against the second-order coefficients of relative peripheral refractive errors. (**A**) Spherical equivalent refraction—a_SER_; (**B**) J_0_ astigmatic component—a_J0_. Home sizes are represented by red triangles and solid lines: < 300 ft^2^; green squares and dotted lines: 300–600 ft^2^; and blue circles and dashed lines: > 600 ft^2^.

Figures 1 and 2 show the relationship between AL/CR and the second-order coefficients of relative peripheral refractive errors, stratified by home sizes. Across all home size groups, children with higher AL/CR exhibited more positive a_SER_, indicating a greater hyperopic shift in the peripheral visual field than those with lower AL/CR (all *p* < 0.001). Notably, the slopes of linear regression lines between AL/CR ratio and a_SER_ were similar across all three home size groups (Fig. 2A). On the other hand, for children living in large homes (> 600 ft^2^), those with lower AL/CR exhibited a more negative a_J0_, indicating a greater against-the-rule astigmatic shift towards the peripheral fields than those with higher AL/CR (Fig. 1B and 2B). The relationship remained significant even after controlling for multiple comparison via Bonferroni adjustment (Table 2). However, for children living in small (< 300 ft^2^) and medium (300–600 ft^2^) homes, the relationships between AL/CR and a_J0_ were insignificant (*p* = 0.40). Our linear regression models confirmed these observations and revealed that home size significantly moderated the relationship between AL/CR and a_J0_, with a significant interaction effect (*p* = 0.02). However, no such interaction effect was found for a_SER_ (*p* = 0.69). A similar trend was also observed in SER (Supplementary Fig. S1), although the results did not reach statistical significance for AL (Supplementary Fig. S2).

## Discussion

The current study investigated the effect of living space and peripheral refractive error on central refractive status (i.e., AL/CR) in a schoolchildren population. Our results found that the relationships between AL/CR and peripheral SER remained relatively consistent across children living in different home sizes. On the other hand, the correlation between AL/CR and peripheral J_0_ was significant only in children residing in large homes. This relationship was abolished in children living in small and medium homes. Our results highlighted a significant interaction between the home size and the relative peripheral astigmatic profile, characterized by the second-order coefficient obtained from quadratically regressing peripheral J_0_.

It has been proposed that the two perpendicular focal planes created by peripheral astigmatism could provide the retina with a cue to differentiate the sign of optical defocus. By comparing the output signal strength of the orientation-tuned neurons, the retina may be able to utilize this signal to direct the eye growth towards the focal plane. Astigmatism is a major component of peripheral refractive error, for which the magnitude could be greater than 10 D at 60° eccentric visual field^31–33^. While most recent studies focused on peripheral SER, few have investigated the relationship between peripheral astigmatism and axial refraction, even rarer in children who are prone to myopia progression. Existing clinical data and animal experiments have demonstrated supportive evidence that uncorrected or lens-induced astigmatism could disrupt the retinal image quality and natural eye-growth process^34–36^. In addition, the peripheral astigmatism was found to be negatively, but weakly, correlated with axial myopia^10^. In a longitudinal study, children who were myopic, as well as those who turned myopic, had less peripheral astigmatism at baseline^37^, suggesting a role of adequate peripheral astigmatism in halting juvenile eye growth. However, the results could not be repeated in a later study^20^. The current study may provide further evidence on the peripheral astigmatic error being a contributing factor to the development of axial myopia^38,39^ in children by revealing a significant relationship between peripheral astigmatism and central refractive error.

As shown in Fig. 1, the refractive profiles shared similar characteristics, with greater AL/CR group differences observed in peripheral SER than in J_0_. As for the relationship with AL/CR, a_SER_ showed a moderate correlation (Table 2), indicating that a more hyperopic peripheral SER may be attributed to posterior stretching during the myopization, as well as the relatively prolate eye shape in myopes^40^. Owing to the independence between AL/CR and a_J0_ in the overall sample, the hypothesis may not be held true that peripheral astigmatism, only when analyzed alone, would provide optical cues to guide the vision-dominated eye growth. Our findings provided insight from the environmental perspective and are discussed in the followings.

Consistent with our previous report on AL and non-cycloplegic SER^8^, a lower AL/CR was also associated with a less constricted living space. Owing to the interrelationship, the peripheral refractive profiles were expected to be more hyperopic in children living in a more constricted space due to a more myopic central refractive error, which was true for peripheral SER (*p* = 0.01, statistics shown in Supplementary Table S3). However, peripheral a_J0_ was independent of home size, as well as having weak correlations with AL/CR regardless of home sizes. As there was a significant interaction effect between peripheral J_0_ profile and home size on AL/CR, it is speculated that the living environment interacted with the peripheral astigmatism and contributed to the central refractive error, of which the effect was also demonstrated in a previous longitudinal study investigating the near work environment at home. This interaction did not appear to come from the direct impact of home size on peripheral astigmatism itself, as both axial and peripheral astigmatisms were similar among all home size groups (Supplementary Table S3). It is deduced that the peripheral astigmatism may be an optical cue for children under a spacious environment with less extrinsic dioptric stimuli. On the other hand, the intrinsic peripheral refractive error, particularly the peripheral astigmatism, had subtle effect for children living in a constricted space, which exerts a greater extrinsic stimulation.

Although accommodation was not pharmacologically controlled by the cycloplegic agent, it is shown to have limited effect on the measurement of the RPRE, which is one of the primary outcomes of this study. Both the central and peripheral fixation targets were positioned equidistant at 6 m away from the eye, only creating a 0.17 D accommodative stimulus at each eccentricity, which was negligible in clinical condition. Assuming that the level of accommodation remained constant during refractive measurement at each eccentricity, it is unlikely to have a significant impact on the RPRE, which is determined by the difference between central and peripheral refractive errors. The refractive status was presented based on the AL/CR ratio, a biometric parameter that is suggested to be independent of the accommodative status^29,30^. The strong correlation between the AL/CR ratio and central M (Supplementary Fig. S3), as consistent with previous epidemiological studies performed under cycloplegic condition^29,41^, also suggested an adequate control of accommodation when performing peripheral refraction. However, the relationship between AL/CR and central SER is non-linear, especially in extreme cases, that careful interpretation of the results is warranted. The primary analysis was based on the quadratic coefficients of peripheral refractive profile obtained by quadratic regressions. Generally, all the fitted curves achieved high R-squares (close to or above 0.90). However, to enhance the representativeness of the fitted curves, it may be needed to increase the number of coordinates (i.e., the number of eccentricities measured across the visual field). Lastly, while home size was the variable of interest in the current study, it may not fully capture the various visual scenes that can impose myopiagenic stimulation on the eye throughout the day^42^. Further studies incorporating personalized sensors may be warranted to observe myopiagenic factors in daily life.

## Conclusions

To conclude, this study provided representative data of the peripheral refractive profile of Hong Kong schoolchildren by adopting a random-cluster sampling, where myopia has reached an epidemic proportion^43,44^, and also revealed a significant interaction between living environment and peripheral astigmatism in relation to axial refractive error in Hong Kong Chinese schoolchildren. Our findings has inputted an environmental factor to evaluate the contribution of peripheral refractive error to the ocular development, as previous researchers have proposed that the retina might distinguish the sign of defocus by comparing orientational input in the periphery^38,39,45^. For instance, a positive defocus, which brings the peripheral astigmatic foci forward, would emphasize the radial component of the retinal image, and increase the retinal signal output as demonstrated in electroretinography studies^25,46^. Further study is needed to understand whether and how other environmental factors, for instance spatial frequency and chromaticity, would contribute to the development and progression of myopia.

## Supplementary Information

Supplementary Information.

## Data Availability

The datasets used and/or analyzed during the current study available from the corresponding author on reasonable request.
