# Supplementary material for: Size of living space as a moderator for central and peripheral refractions in children
Source: Sci Rep. 2023 Jul 4;13:10797. doi: 10.1038/s41598-023-37454-w (PMC10319720; doi:10.1038/s41598-023-37454-w)
Supplement: Supplementary file 1 — Supplementary Information. [file 41598_2023_37454_MOESM1_ESM.docx]

Supplementary Table S1. Regression and correlation statistics stratified by home size groups for SER.

|  |  | **Home size (ft^2^)** | | |  |
| --- | --- | --- | --- | --- | --- |
|  | **All** | **<300** | **300 – 600** | **>600** |  |
|  | *Regression coefficients – Unstandardized B (95% confidence interval)* | | | | *Moderator t (p)* |
| **a_SER_** | -0.54 (-0.62, -0.47) | -0.57 (-0.75, -0.39) | -0.56 (-0.67, -0.46) | -0.39 (-0.50, -0.28) | 1.09 (0.28) |
| **a_J0_** | -0.22 (-0.39, -0.05) | -0.14 (-0.48, 0.20) | -0.14 (-0.38, 0.10) | -0.47 (-0.70, -0.23) | **-2.01 (0.04)** |
|  | *Partial correlation coefficients – r (p)* | | | | *χ^2^ (p)* |
| **a_SER_** | -0.50 (<0.001) | -0.44 (<0.001) | -0.52 (<0.001) | -0.57 (<0.001) | 2.13 (0.35) |
| **a_J0_** | -0.11 (0.01) | -0.06 (0.42) | -0.07 (0.26) | -0.37 (<0.001) | **8.55 (0.01)** |

*Bolded text indicates significant interaction effect*

Supplementary Table S2. Regression and correlation statistics stratified by home size groups for AL.

|  |  | **Home size (ft^2^)** | | |  |
| --- | --- | --- | --- | --- | --- |
|  | **All** | **<300** | **300 – 600** | **>600** |  |
|  | *Regression coefficients – Unstandardized B (95% confidence interval)* | | | | *Moderator t (p)* |
| **a_SER_** | 0.20 (0.15, 0.25) | 0.15 (0.04, 0.26) | 0.23 (0.16, 0.30) | 0.19 (0.10, 0.27) | 0.74 (0.46) |
| **a_J0_** | 0.13 (0.04, 0.23) | 0.06 (-0.14, 0.25) | 0.12 (-0.02, 0.26) | 0.26 (0.09, 0.43) | 1.57 (0.12) |
|  | *Partial correlation coefficients – r (p)* | | | | *χ^2^ (p)* |
| **a_SER_** | 0.32 (<0.001*)* | 0.19 (0.01) | 0.36 (<0.001) | 0.39 (<0.001) | 4.30 (0.12) |
| **a_J0_** | 0.11 (0.01) | 0.04 (0.58) | 0.10 (0.10) | 0.29 (0.003) | 4.34 (0.11) |

*Bolded text indicates significant interaction effect*

Supplementary Table S3. Second-order coefficients of peripheral refraction among home size groups. Data are presented in mean ± standard deviation.

|  | Home size | | | Age-controlled ANCOVA F (p) |
| --- | --- | --- | --- | --- |
|  | <300 ft^2^ | 300 – 600 ft^2^ | >600 ft^2^ |  |
| a_SER_ (x10^-3^) | 0.41 ± 1.45 | 0.19 ± 1.61 | -0.18 ± 1.64 | 4.55 (0.01)* |
| a_J0_ (x10^-3^) | -1.19 ± 0.85 | -1.16 ± 0.82 | -1.28 ± 0.89 | 0.75 (0.47) |

*Asterisks indicate significant difference between <300 ft^2^ and >600 ft^2^ groups after Bonferroni adjustment.


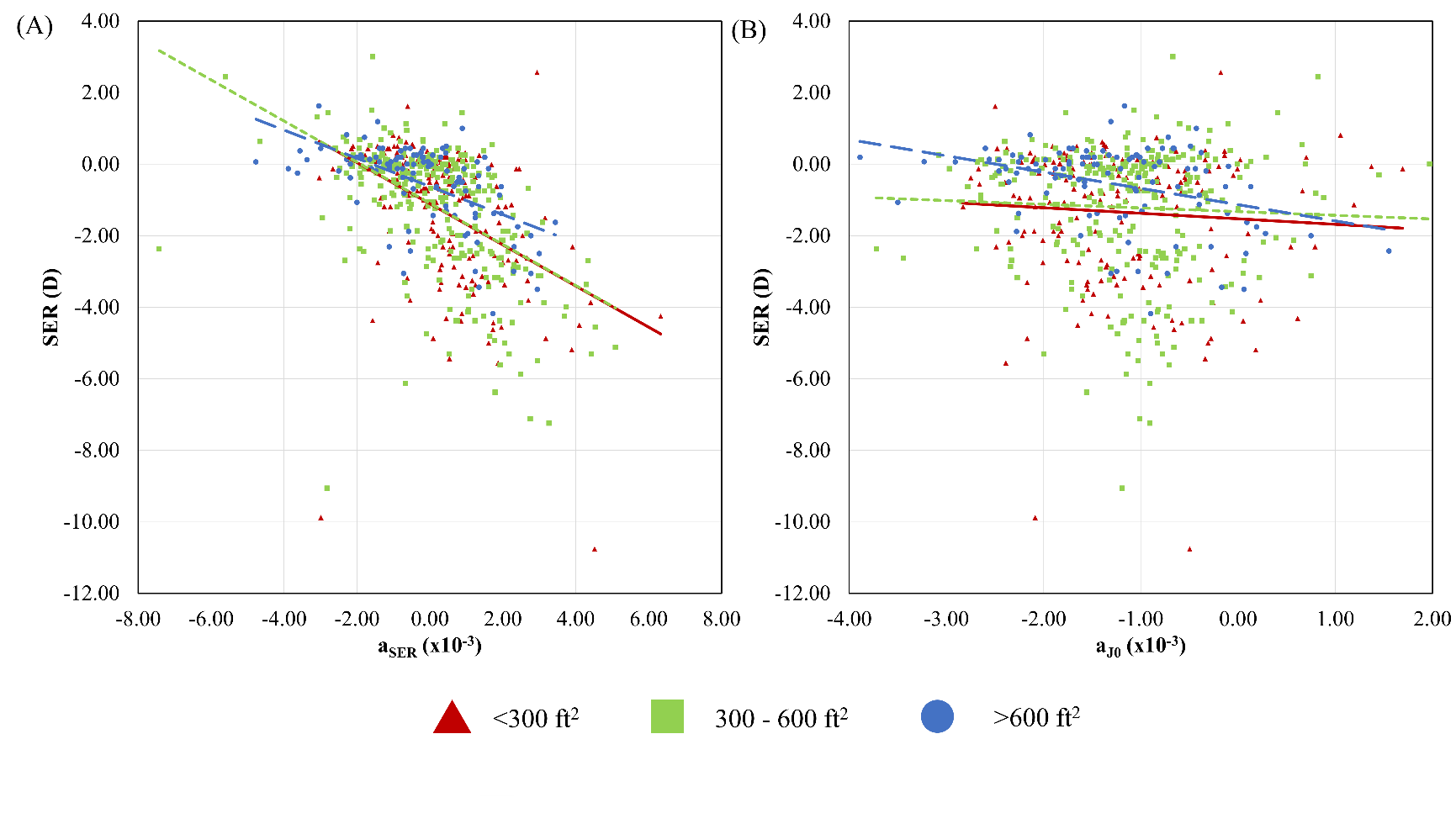


Supplementary Figure S1. Relationships between SER against the second-order coefficients of relative peripheral refractions. (A) Spherical equivalent refraction – a_SER_; (B) J_0_ astigmatic component – a_J0_. Home sizes are represented by red triangles and solid lines: <300 ft^2^; green squares and dotted lines: 300 – 600 ft^2^; and blue circles and dashed lines: >600 ft^2^.


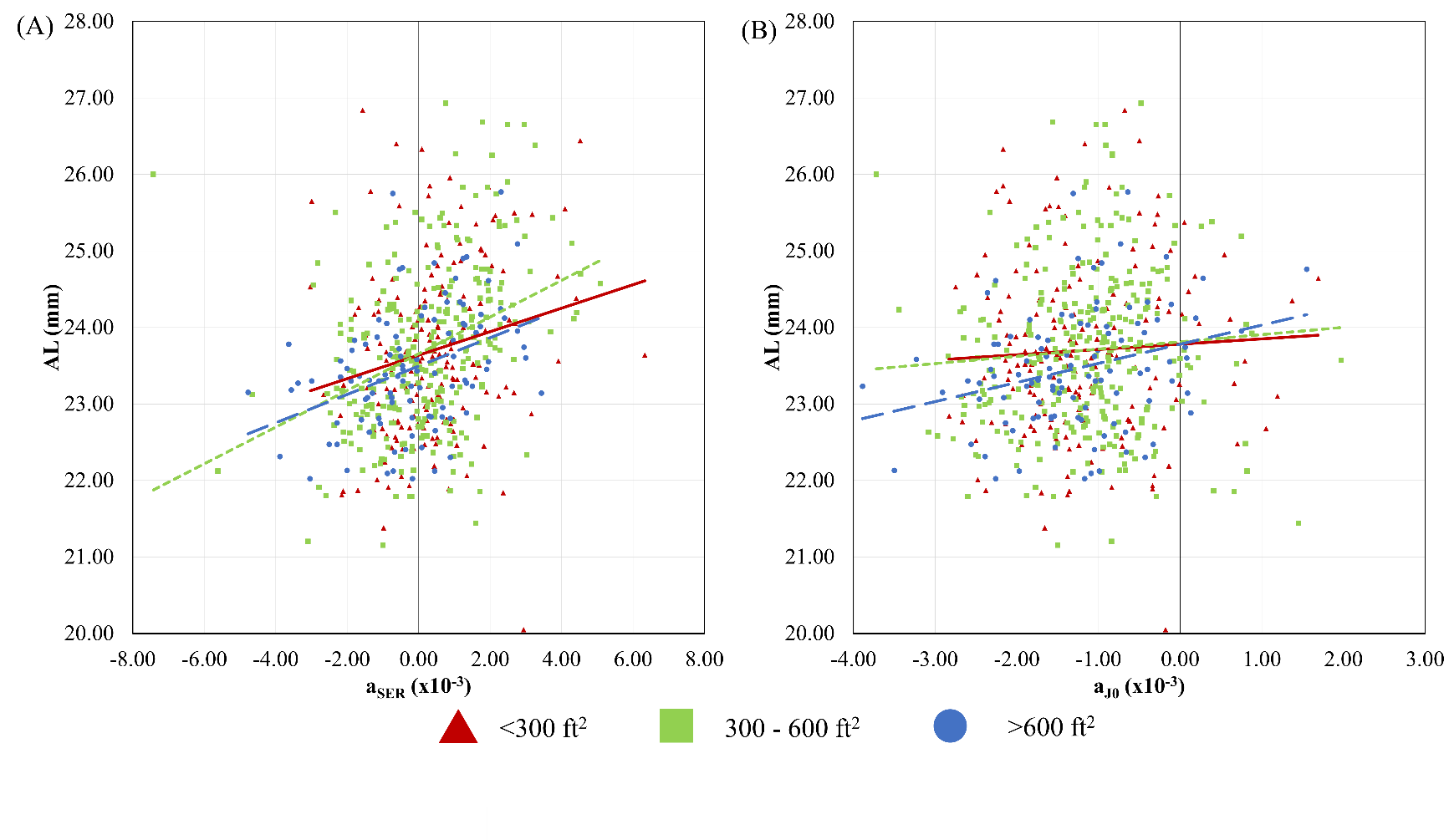


Supplementary Figure S2. Relationships between AL ratio against the second-order coefficients of relative peripheral refractions. (A) Spherical equivalent refraction – a_SER_; (B) J_0_ astigmatic component – a_J0_. Home sizes are represented by red triangles and solid lines: <300 ft^2^; green squares and dotted lines: 300 – 600 ft^2^; and blue circles and dashed lines: >600 ft^2^.


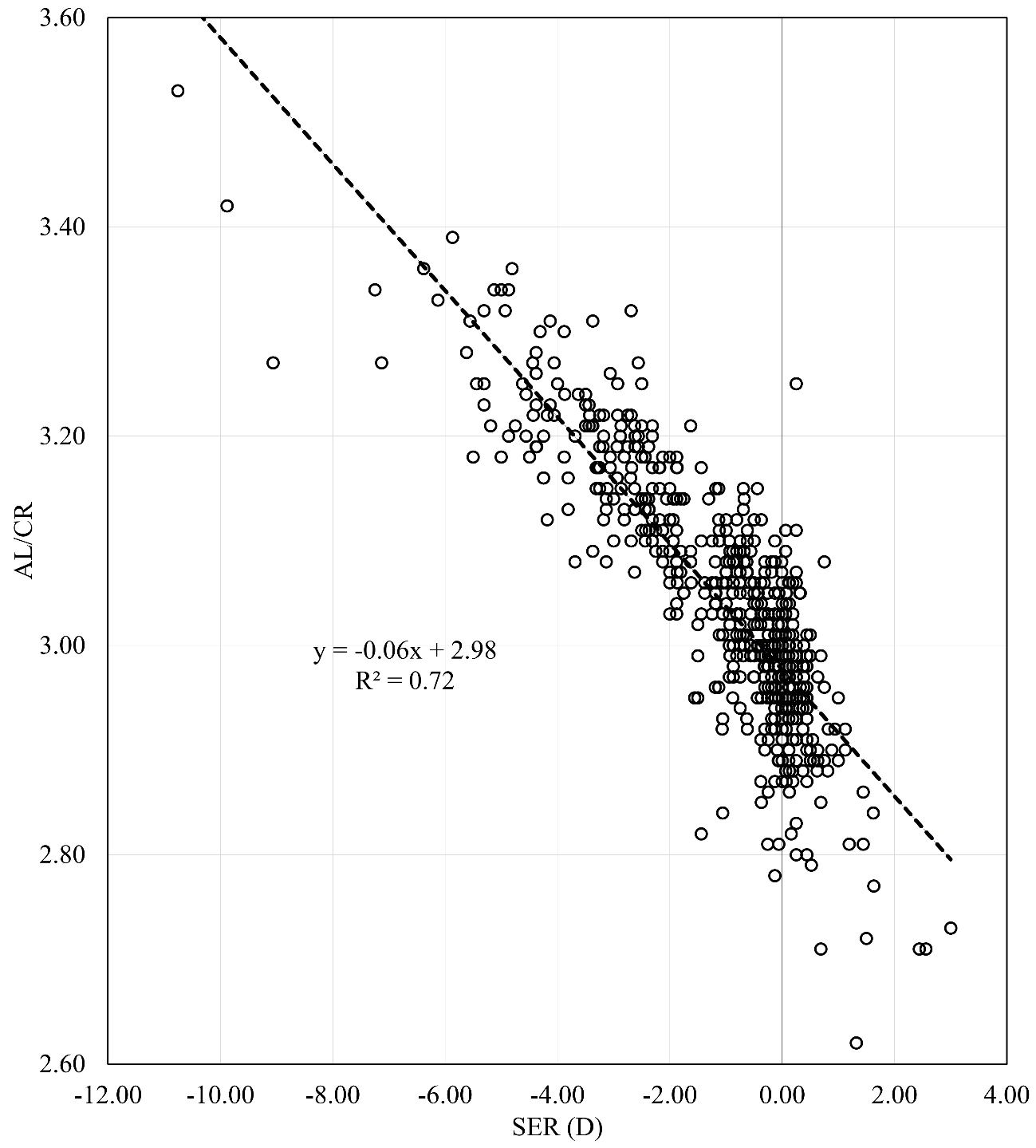


Supplementary Figure S3. Correlation between SER and AL/CR ratio. Linear regression equation: $AL/CR=-0.06\left( SER \right)+2.98$
